# Intracerebral EEG Artifact Identification Using Convolutional Neural Networks

**DOI:** 10.1007/s12021-018-9397-6

**Published:** 2018-08-13

**Authors:** Petr Nejedly, Jan Cimbalnik, Petr Klimes, Filip Plesinger, Josef Halamek, Vaclav Kremen, Ivo Viscor, Benjamin H. Brinkmann, Martin Pail, Milan Brazdil, Gregory Worrell, Pavel Jurak

**Affiliations:** 1 International Clinical Research Center, St. Anne’s University Hospital, Brno, Czech Republic; 2 The Czech Academy of Sciences, Institute of Scientific Instruments, Brno, Czech Republic; 3 Department of Neurology, Mayo Clinic, Mayo Systems Electrophysiology Laboratory, Rochester, MN USA; 4 Brno Epilepsy Center, Department of Neurology, St. Anne’s University Hospital and Medical Faculty of Masaryk University, Brno, Czech Republic; 5 CEITEC – Central European Institute of Technology, Masaryk University, Brno, Czech Republic; 6 Department of Physiology and Biomedical Engineering, Mayo Clinic, Rochester, MN USA

**Keywords:** Intracranial EEG (iEEG), Noise detection, Convolutional neural networks (CNN), Artifact probability matrix (APM)

## Abstract

Manual and semi-automatic identification of artifacts and unwanted physiological signals in large intracerebral electroencephalographic (iEEG) recordings is time consuming and inaccurate. To date, unsupervised methods to accurately detect iEEG artifacts are not available. This study introduces a novel machine-learning approach for detection of artifacts in iEEG signals in clinically controlled conditions using convolutional neural networks (CNN) and benchmarks the method’s performance against expert annotations. The method was trained and tested on data obtained from St Anne’s University Hospital (Brno, Czech Republic) and validated on data from Mayo Clinic (Rochester, Minnesota, U.S.A). We show that the proposed technique can be used as a generalized model for iEEG artifact detection. Moreover, a transfer learning process might be used for retraining of the generalized version to form a data-specific model. The generalized model can be efficiently retrained for use with different EEG acquisition systems and noise environments. The generalized and specialized model F1 scores on the testing dataset were 0.81 and 0.96, respectively. The CNN model provides faster, more objective, and more reproducible iEEG artifact detection compared to manual approaches.

## Introduction

In general, EEG artifacts and undesired signals can be generated by biological phenomena (eye blinks, head movement, muscle activity, cardiac signals), acquisition instrumentation (signal discontinuities, transient filter effects), or external sources (electromagnetic inductive noise). Automated detection and removal of scalp EEG artifacts have been widely explored. Scalp EEG artifacts can be detected by signal processing and statistical methods (Delorme et al. 2007; Gerla et al. 2017), wavelet transform time-frequency methods (Bern 2015; Kiamini et al. 2008; Huang et al. 2014), Hilbert-Huang transform (Yan et al. 2008; Wang and Su 2014), spatio-temporal signal processing (Liu and Yao 2006) (in cases were the electrode spatial information is known), adaptive filtering (Somers and Bertrand n.d.), independent component analysis (Islam et al. 2016; T. Radüntz et al. 2015; Delorme et al. 2007), or machine learning methods (Thea Radüntz et al. 2017).

However, methods for automatic detection of artifacts that occur in intracerebral EEG (iEEG) have received less attention. Historically, iEEG recordings were assumed to be largely, immune to eye movement and muscle artifacts. This assumption has more recently been proven incorrect (Ball et al. 2009; Jerbi et al. 2009; Kovach et al. 2011) and there is now a generally recognized need for automated, unbiased, methods to remove iEEG artifacts (Hu et al. 2007), particularly in wide-bandwidth (Worrell et al. 2012) and microwire recordings (Stead et al. 2010). In general, iEEG can be contaminated by similar artifact sources to scalp EEG, but the effect tends to be weaker than in the scalp electrodes and may be predominantly present in contacts located near the scalp or near cranial nerve foramen (muscle artifacts, eye movements, electrocardiographic signals). Scalp EEG artifacts might also be transferred to intracranial electrodes by use of a scalp or epidural common reference. There also exist artifacts introduced specifically by iEEG instrumentation (subdural and depth), such as movement of electrode in the tissue (e.g. by natural pulsatile movement of the brain).

**Fig. 1:**
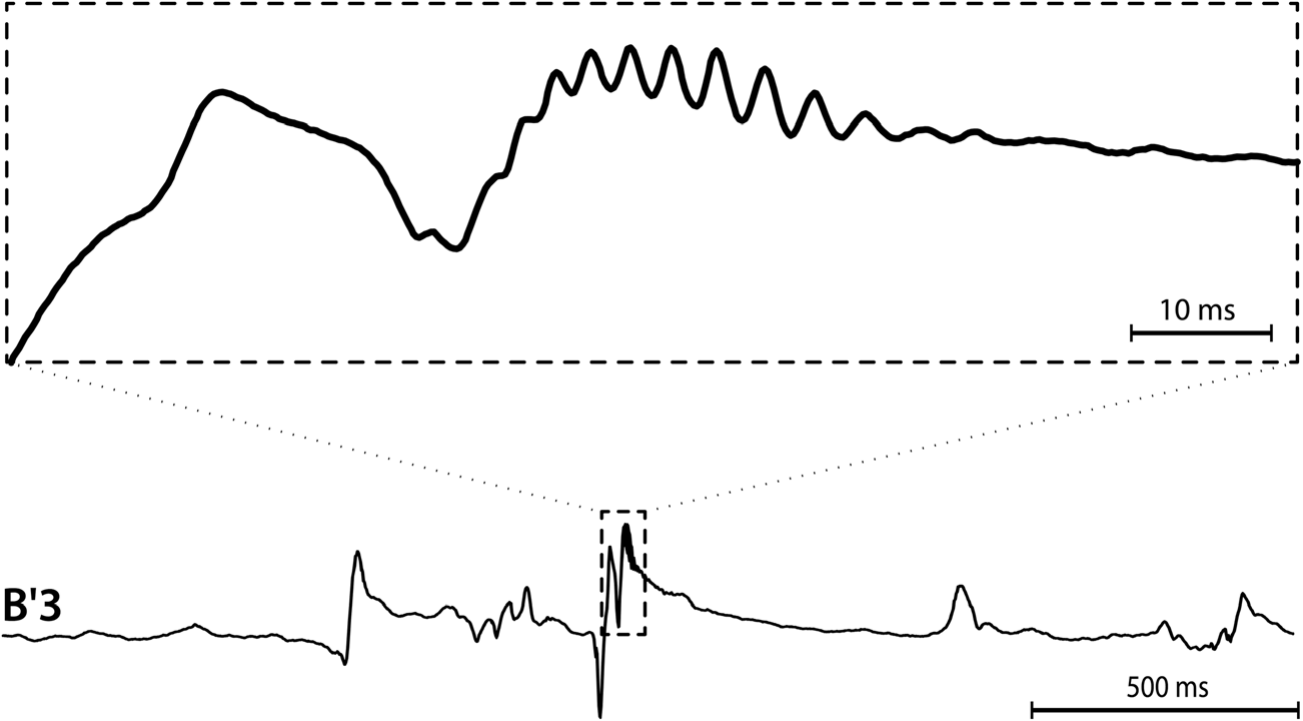
Example of pathological activity seen in epileptogenic brain, and characterized as a sharp wave transient. The blow up of the pathological transient shows a high frequency oscillation (HFO) riding on spike peak

The primary application of iEEG is evaluation of drug resistant epilepsy (DRE). Therefore, the electrodes are often placed in pathological brain tissue generating pathological activity. Hence the iEEG recording contains both pathological and physiological activity (Fig. 1), and the pathological activity (e.g. interictal spikes and high-frequency activity) share some characteristics with common artifacts. Most iEEG signal processing studies use expert visual review or simple automated artifact rejection approaches. For example a method for detection of noise-free segments based on estimation of line length in bandpass filtered data was published in (Gliske et al. 2016), however detector performance (Recall and PPV) is not specified. More sophisticated techniques using independent component analysis for removing of scalp reference artifacts from iEEG signals have been explored but are not comprehensively validated (Hu et al. 2007). Recording iEEG over multiple days with high sampling rates (1 kHz to 32 kHz) and high channel counts (256) creates large datasets that make expert visual scoring nearly impossible. Automation and usage of modern deep-learning techniques with strong pattern recognition capabilities may be capable of providing an accurate, automated method for iEEG artifact identification.

Convolutional neural networks (CNN) were originally designed for computer vision and character recognition by Lecun (Lecun and Bengio 1995). Since their invention, CNNs have gained in effectiveness and popularity, driven by increasing computing power and graphical processing unit (GPU) advances. In recent years, deep learning techniques have been shown to significantly improve classification tasks in the scientific and industrial fields (Krizhevsky et al. 2012). In comparison with traditional machine learning techniques convolutional neural networks (CNNs) do not require manual feature extraction. Moreover, CNNs exhibits translational invariance, which gives them ability to localize given patterns independently on location in the given image. In general, we can use this technique for classification of time-series. Time-series might be treated as 1D image or some time-frequency transformations (Fourier transform or Wavelet transform) might be used for transformation from signal to image. Lastly, CNNs were also proven to work with 1D signals in fields like speech recognition (Zhang et al. 2017). Regarding given facts, CNNs exhibits strong potential to be used in biological signal processing.

More recently, CNNs have been used in biological time series signal processing, including Electrocardiogram (Kiranyaz et al. 2016; Rahhal et al. 2016), Polysomnography (Supratak et al. 2017), and electroencephalography (Schirrmeister et al. 2017a, b). Unlike other machine learning algorithms that use EEG frequency, spatial and temporal features as inputs, CNNs can learn directly from the data without the need for feature identification and extraction, which can be time consuming and may introduce bias into the machine learning model. The downside of CNN methods has been the need for large amounts of expert-annotated data required for training. Given the excellent performance by CNNs in similar applications it seems reasonable to assess the capability of these networks for rejecting recording artifacts in iEEG data.

The aim of this study is to develop and validate convolutional neural networks (CNN) for fully automated iEEG noise detection. This research is focused on supporting detection of high frequency oscillations (HFO) and localization of epileptiform activity. For this reason, this method must avoid mutual misclassification between pathological activity and artifacts. Therefore, this algorithm is designed to meet the condition. The proposed method automates, labelling artifacts and noise in iEEG, and can significantly improve pre-processing in large studies with high sampling rates and channel counts. The proposed method not only eliminates the subjectivness of expert interpretation, but is also significantly faster. Here we describe a method that provides independent detections for each channel separately and generates an artifact probability matrix (APM) that offers visual feedback of automated detections. Lastly, we demonstrate the method can be generalized to other iEEG acquisition systems with a limited amount of additional training data to characterize the acquisition system specific artifacts.

## Material and Methods

### Data Recording

We retrospectively analyzed iEEG data from the Department of Neurology, St. Anne’s University Hospital (Brno, Czech Republic) and Mayo Clinic (Rochester, Minnesota, U.S.A). Data from St. Anne’s University Hospital consisted of iEEG recordings obtained from 11 patients with DRE who were undergoing evaluation for epilepsy surgery. All patients were implanted with standard intracranial depth electrodes (5, 10 and 15 contact semi-flexible multi-contact platinum electrodes (ALCIS), with a diameter of 0.8 mm, a contact length of 2 mm, contact surface area 5.02 mm^2^ and inter-contact distance 1.5 mm). A 192-channel research EEG acquisition system (M&I; Brainscope, Czech Republic) with 25 kHz sampling rate and common reference montage was used to record 30 min of awake resting interictal iEEG recordings used in this study. Raw data was filtered in the bandwidth of 2 kHz, and downsampled to 5 kHz. The present study is carried out in accordance with the ethical standards and the study procedures were approved by St. Anne’s University Hospital Research Ethics Committee and the Ethics Committee of Masaryk University. All subjects gave written informed consent in accordance with the Declaration of Helsinki.

Data from Mayo Clinic consisted of two-hour long iEEG recordings obtained from 25 patients with DRE undergoing evaluation for epilepsy surgery. Two hour data segments were taken from the first night of patient’s stay at ICU between 1 AM and 3 AM. Data was acquired using a Neuralynx Cheetah system (Neuralynx Inc., Bozeman MT) and sampled at 32 kHz with hardware filter bandwidth of DC – 9 kHz. Subsequently, an antialiasing filter was applied to the data (Bartlett-Hanning window, 1 kHz), and data was downsampled to 5 kHz. Patients were implanted with depth electrodes (AD-Tech Medical Instrument Corp., Racine, WI or PMT, Chahassen, MN). Electrodes consisted of 4 or 8 Platinum/Iridium contacts (2.3 mm long, 1 mm diameter, spaced 5 or 10 mm center-to-center). Subdural grids and strips are composed of 4.0 mm diameter Platinum/Iridium discs (2.3 mm exposed) with 10 mm center-to-center distance. This study was carried out in accordance with the recommendations of the Mayo Clinic Institutional Review Board with written informed consent from all subjects. All subjects gave written informed consent in accordance with the Declaration of Helsinki. The protocol was approved by the Mayo Clinic Institutional Review Board.

### Manual Artifact Detection Technique

**Fig. 2:**
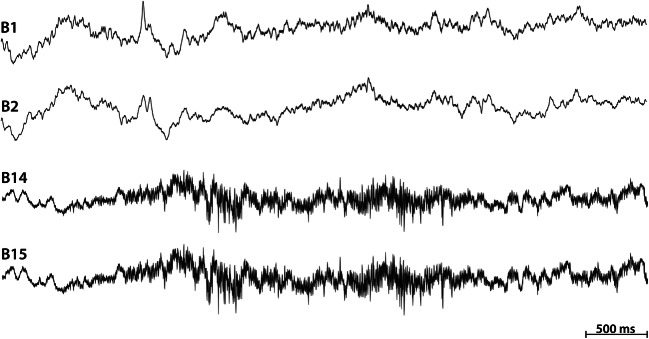
Example of physiological iEEG in channels B1-B2, compared to muscle

We used a previously published (Brázdil et al. 2017) manual detection technique for expert artifact annotations. A key aspect for the detection of artifacts were the power envelopes (envelograms) in several frequencies bands and calculation of power distribution matrices (PDM). PDMs were visually inspected to identify artifact candidates, and these areas were manually verified in the raw iEEG data. Although the PDM allows inspection of all channels in a single image, subsequent verification in raw signals was also performed. Figure 1 shows example epileptogenic pathological activity in single channel, while Fig. 2 compares two channels with and two without artifactual segments, in this case showing motion and muscle artifacts.

artifacts present at same time in channels B14 and B15.

### Datasets and Classes

All recorded data were used to create a labelled dataset for future machine learning experiments. All 11 datasets from St. Anne’s University Hospital (Czech Republic) and 25 datasets from Mayo Clinic (USA) were manually annotated. Artifacts and pathological activity were annotated in each individual channel separately. Signals were examined and annotated in SignalPlant, a free software tool for signal inspection and processing (Plesinger et al. 2016). Data were manually scored into one of five classes (*Physiological iEEG*, *Pathological iEEG*, *50 Hz Line noise*, *60 Hz Line noise*, and *Non-cerebral artifact*) and subsequently segmented into segments of 3-s length (15,000 samples). All data segments containing pathological activity (including interictal spikes, high frequency oscillations) were set to *Pathological iEEG*. Segments with power line interference (50 Hz or 60 Hz) were set to the *Line Noise* class. All segments representing non-cerebral activity (e.g. muscle, movement, machine artifacts etc.) were assigned to *Non-cerebral artifacts*. The remaining training data segments were automatically assigned to *Physiological iEEG*. The final number of annotated examples for each class is shown in Table 1. A selected segment length of 3 s was heuristically set to account for the fact that muscle artifacts may span several seconds. The dataset from Saint Anne’s hospital was then split into training (70% of each class) and validation (30% of each class) data, and the Mayo Clinic dataset was used for testing (Fig. 4).

**Table 1:** Number of 3-s length segments from St. Anne’s University Hospital and Mayo Clinic in each class based on manual scoring by experts

	St. Anne’s University Hospital	Mayo Clinic
Classification category	Segments in category	Segments in category
Physiological iEEG	66,581	44,259
Pathological iEEG	18,184	6099
Noise and muscle activity	13,777	25,389
Power line noise (50hz/60hz)	13,825	22,420
Total	112,367	98,167

### Data Preprocessing

**Fig. 3:**
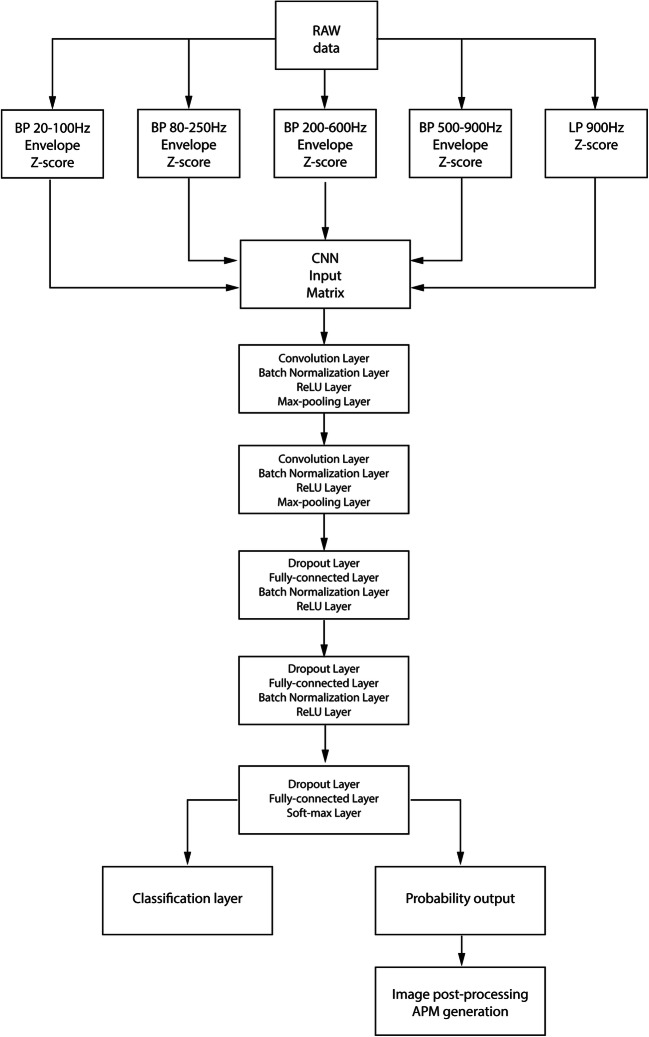
Flowchart of the CNN system. As an input of the CNN a z-score of raw data for each 3-s epoch was used as well as envelograms in five different frequency bands. Drop out layers, Batch Normalization Layers and L2 regularization is used to control for over-training. The Artefacts Probability Matrix (APM) was generated from a resulting image

We constructed a CNN with a matrix input layer (5 × 15000 samples). The first row of the input matrix was a low-pass filtered raw data segment (cutoff frequency 900 Hz). Subsequent input rows consist of the following bandpass envelograms: 20–100 Hz, 80–250 Hz, 200–600 Hz, 500-900 Hz (Fig. 3). Envelograms were computed from bandpass filtered signals using the squared absolute value of the Hilbert transformed signal. For the lowpass and bandpass filtering, we used 3rd order Butterworth zero-phase filters. Each row of matrix was normalized using a z-score to form CNN inputs. Envelogram data were used to provide more specific information about the frequency distribution of data for CNN. Data ranges were selected to account for several physiological bands in known frequency distribution of iEEG (20–100 Hz spanning beta, low and high gamma activity, 80–250 Hz ripples, 200–600 Hz fast ripples and 500–1000 Hz very fast ripples) as well as for muscle and motion artifacts.

### Architecture of Convolutional Neural Network and Training Methods

The CNN is a modification of a feed-forward neural network that uses weight sharing and exhibits translation invariance. Learning in the CNNs operates on the same principle as a traditional feed-forward neural network where an error from output layer is back-propagated through the network and weights of the network are proportionally updated to the gradient of error. More sophisticated descriptions can be found in (LeCun 1988, 2015; LeCun and Kavukcuoglu 2010; Lecun and Bengio 1995). The architecture of CNN typically consists of convolutional layers, batch normalization layer, nonlinearity mapping layer and pooling layer. These layers are stacked several times on each other to form complex feature extraction module. Extracted features from convolutional layers are propagated through fully-connected layers to perform classification or regression (depending on the task). Over-fitting of CNN is controlled by dropout layers and L2 regularization.

**Fig. 4:**
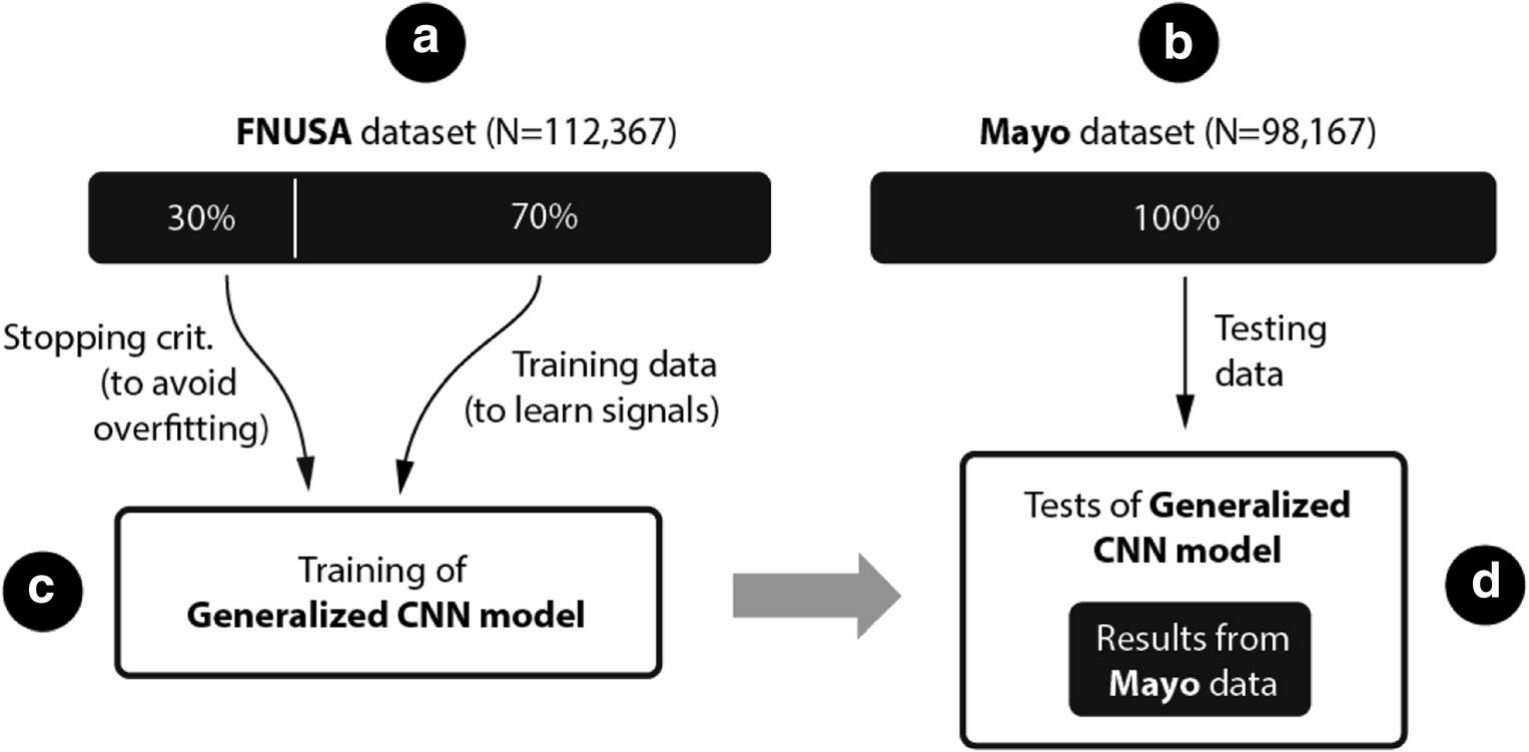
Flowchart illustrating training for generalized model. A- St. Anne’s University Hospital (FNUSA) dataset (Table 1), B- Mayo clinic dataset (Table 1), C- training of generalized model, D- generalized model results for out of institution/out of patient testing Mayo Clinic dataset (Table 3)

To create a generalized model (GM), we have used training (70%) and validation data (30%) from St. Anne’s University Hospital. Results for generalized model are reported based on out of institution testing (Mayo Clinic). The generalized model was trained for classification into 3 groups: Noise and muscle activity, Physiological iEEG, and Pathological iEEG. The power line noise group was excluded due to power line frequency differences between the EU (50 Hz) and USA (60 Hz). Classification of powerline noise is introduced by retraining of generalized model with small part of Mayo Clinic dataset (training data) to form a specific model for Mayo Clinic data. The setup of the CNN network is shown on Fig. 3. The CNN was built and trained in Matlab 2017b extended by the parallel computing toolbox, neural network toolbox, statistics and machine learning toolbox. The generalized model (GM) was trained using training St. Anne’s University Hospital data, and validation data (out of sample) were used to avoid overtraining and to stop the training process (network weights are not updated during validation process). This way, the GM was trained until the performance on validation data started to decline or reached 25 training epochs. Complete Mayo dataset was used for model testing. This provides a worst case scenario for classification, where model is trained on different data than testing data obtained from another acquisition system and under diverse measurement conditions. Results for the trained GM are reported for cross-institution testing based on Mayo Clinic dataset (Fig. 4).

**Fig. 5:**
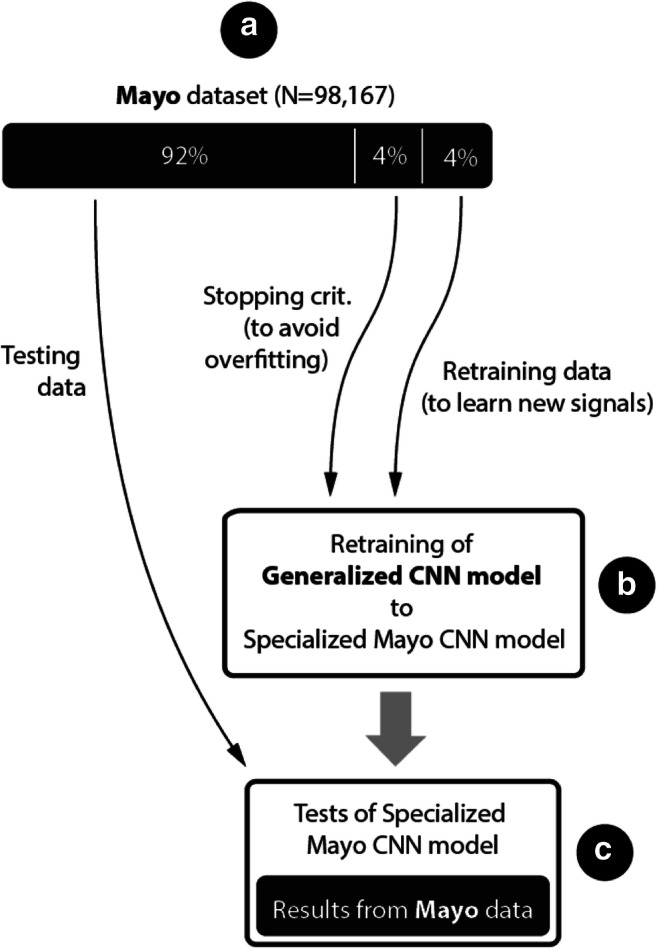
Flowchart illustrating training for specialized model.A- Mayo clinic dataset (Table 1), B- retraining of generalized model, C- generalized model results for out of sample testing Mayo Clinic dataset (Table 5)

For transfer learning (Fig. 5) we used the trained GM and Mayo training data to generate a specialized model for Mayo data, including 60 Hz power line noise. During the transfer learning process, the last fully connected layer with softmax activation function was reset to a random state with a normal distribution. Next, we retrained the model with the Mayo training dataset, with the learning rate of the transferred layers set to 10% of the learning rate of last fully connected layer with softmax activation function. Mayo clinic dataset previously used for testing of GM was divided into small training (4%), small validation (4%) and testing dataset (92%) and used for out of sample model training.

### Results

Precision, recall, and F1 scores are reported below for evaluation of the proposed machine learning method with an unbalanced dataset. (each category has a different number of observations). The confusion matrix (Table 2) shows the classification results of GM trained on St. Anne’s University Hospital data and tested on the complete Mayo Clinic dataset (Table 3). The confusion matrix (Table 4) shows transfer learning results after retraining the GM on Mayo training data to be specialized for Mayo Clinic data. Note that we have used only 4% of randomly selected data from Mayo Clinic as training dataset and another 4% as validation dataset for the transfer learning process and rest of data was used as testing dataset. The resulting average F1-scores for GM and the specialized model were *F*_1_ = 0.81 and *F*_1_ = 0.96, respectively. Results for Recall, positive prediction value (PPV) and F1 scores for the generalized and specialized models are shown in Table 3, Table 5.

**Table 2:** A confusion matrix of the generalized iEEG model (trained on St Anne’s University Hospital training dataset). Tensting was performed on complete Mayo Clinic dataset

	Automated Classification				
Gold Standard		Noise and muscle activity	Physiological iEEG	Pathological iEEG	Total
	Noise and muscle activity	23,253	1241	895	25,389
	Physiological iEEG	3073	38,647	2539	44,259
	Pathological iEEG	20	1549	4530	6099
	Total	26,346	41,437	7964

**Table 3:** Classification results of the generalized iEEG model (trained on St Anne’s University Hospital training dataset). Testing was performed on Mayo Clinic testing dataset

Classification Category	Recall	PPV	F1
Noise and muscle activity	0.91	0.88	0.89
Physiological iEEG	0.87	0.93	0.90
Pathological iEEG	0.74	0.56	0.64
Average	0.863	0.804	0.81

**Table 4:** A confusion matrix of the specialized Mayo Clinic data model (generalized model retrained by small Mayo Clinic training dataset). Testing was performed on Mayo Clinic testing dataset

	Automated Classification					
Gold Standard		Power line noise (60hz)	Noise and muscle activity	Physiological iEEG	Pathological iEEG	Total
	Power line noise (60hz)	19,949	171	487	19	20,626
	Noise and muscle activity	44	23,084	183	47	23,358
	Physiological iEEG	87	531	39,625	475	40,718
	Pathological iEEG	0	29	454	5128	5611
	Total	20,080	23,815	40,749	5669

**Table 5:** Classification results of the specialized Mayo Clinic data model (generalized model retrained by small Mayo Clinic training dataset). Testing was performed on Mayo Clinic testing dataset

Classification Category	Recall	PPV	F1
Power line noise (60hz)	0.96	0.99	0.98
Noise and muscle activity	0.98	0.96	0.97
Physiological iEEG	0.97	0.97	0.97
Pathological iEEG	0.91	0.90	0.90
Average	0.96	0.95	0.96

### Artifact Probability Matrix and Pathology Probability Matrix

**Fig. 6:**
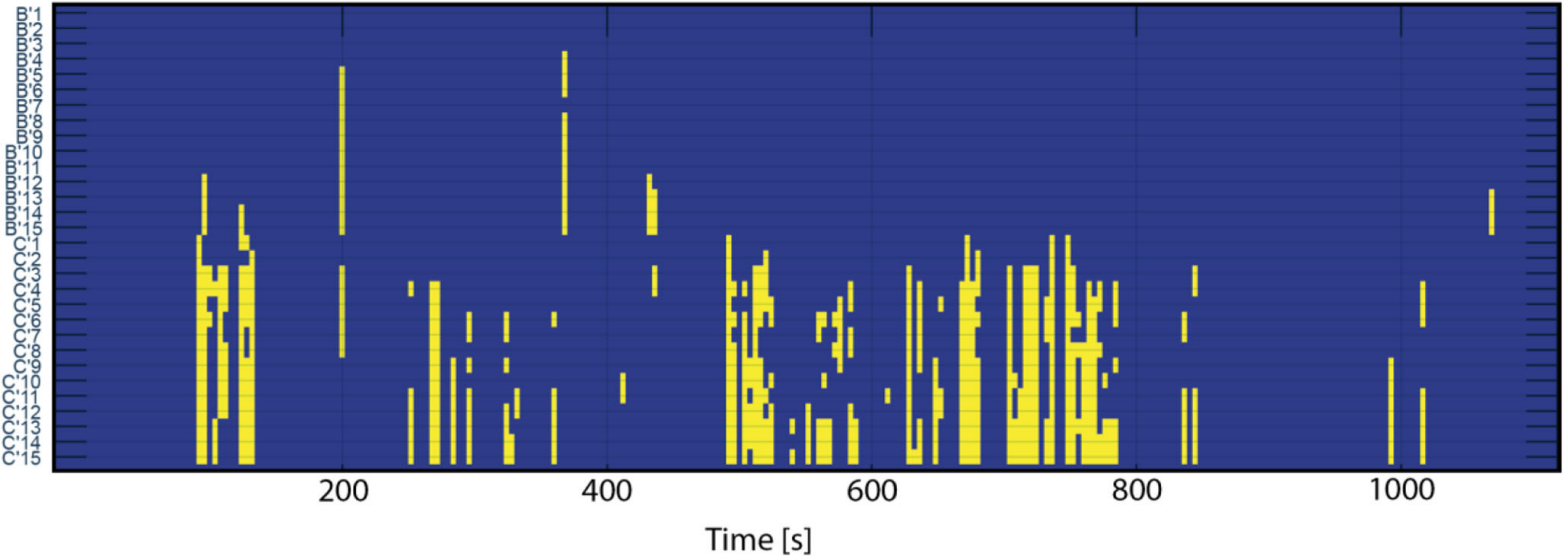
Example of binarized Artifacts Probability Matrix (APM). Yellow stripes indicate artifacts with a probability higher than 95%. Y-axis shows iEEG channels and X-axis shows time in seconds

**Fig. 7:**
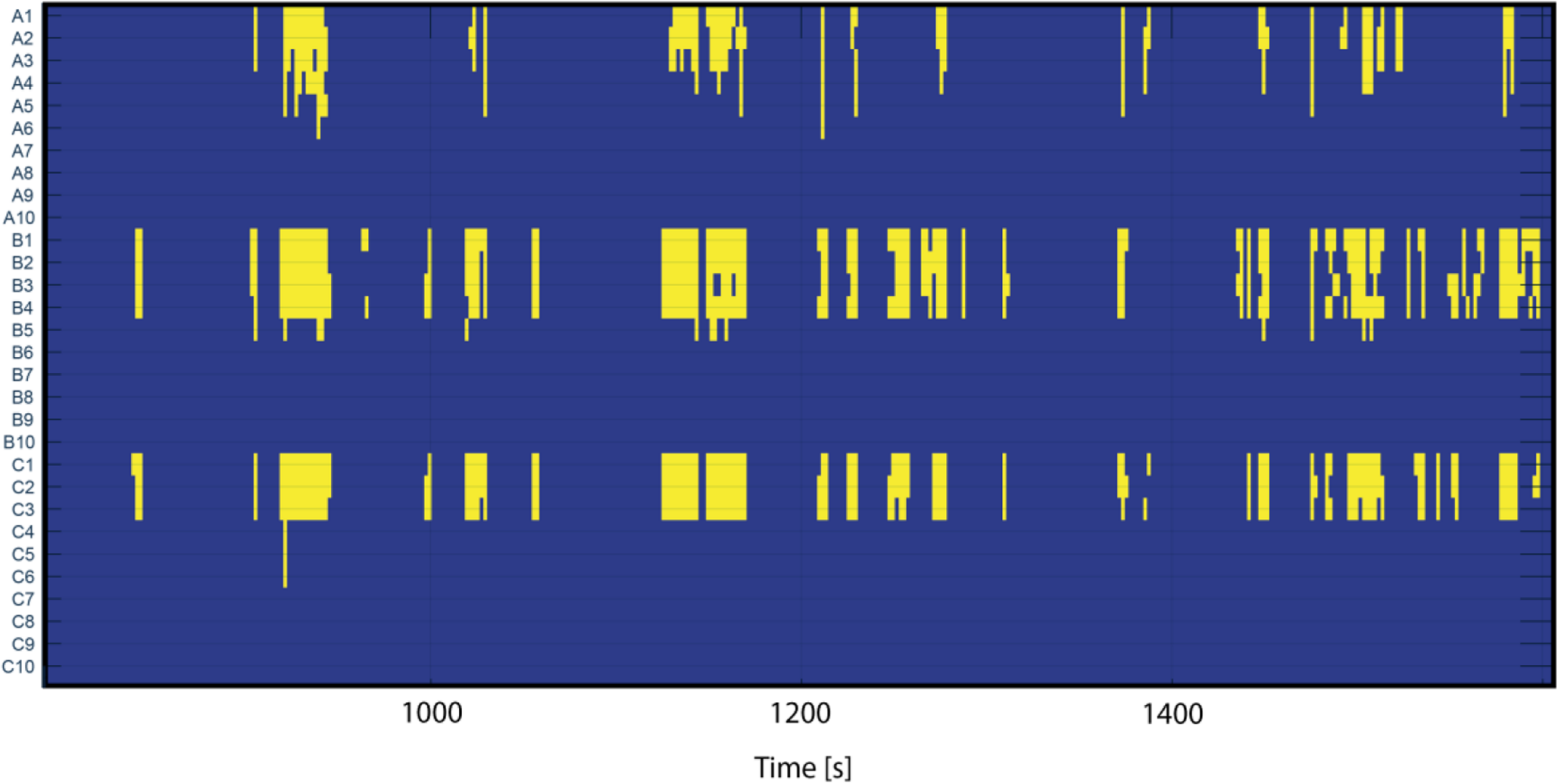
Example of binarized pathology probability matrix (PPM). Yellow stripes indicate patology segments with a probability higher than 95%.Y-axis shows iEEG channels and X-axis shows time in seconds

In order to provide a graphical method for interpretation of results, we generated an Artifact Probability Matrix (APM, Fig. 6) and a Pathology Probability Matrix (PPM, Fig. 7) as an alternative to the PDM discussed in (Brázdil et al. 2017). Matrices consist of blue background with yellow dots (stripes) that indicate a probability higher than 95% of artifact or pathology in the corresponding iEEG data segment. Each row denotes a different electrode and each column a 1-s iEEG epoch. The CNN model classifies a 3 s segment with overlap of 2 s, and the probability is assigned to the center 1 s span of the segment. This overlap method is used in order to make sure that algorithm does not miss events localized between two windows. Areas classified as artifacts can be easily localised by visual inspection of APM or by fully-automatic image processing procedures. The fully-automatic method can for example use connected component labelling and morphological operations on the image to select most significant artifact or pathological data segments in iEEG record. Because all automated detections are electrode specific, all detections in each channel are independent from other channels.

## Discussion

We trained a generalized CNN model with a training dataset from one institution and tested on a separate dataset from another institution. We showed that a generalized CNN model can be used for iEEG classification with data acquired by different acquisition systems with different parameters of measurements. Most importantly, the general model could be optimized using a relatively small amount of data from the second acquisition system. This produced better performance of the model that was retrained by a small amount of labelled data (4%) from the new institution. Conventionally, CNNs require large datasets for training, and this can be a significant limitation if the training data requires time consuming annotation of the primary data, as in the case here of iEEG. Here we show that after training the GM using a large initial training dataset, the CNN can be retrained for a different acquisition system (Neuralynx, Inc.) and recording environment (Mayo Clinic, USA) with modest data requirements. By training the generalized model on data from St. Anne’s University Hospital, and enhancing and retraining it with a relatively small amount of labelled Mayo data, we obtained a specific generalized model using a small amount of iEEG data measured under different conditions and acquisition system. This approach achieved a mean F1 score of 0.81 with the generalized CNN model and 0.96 with the Mayo Clinic data specialized model.

The resulting solution for automated labelling of large datasets provides objective and fast artifact detection compared to manual approaches. The method used feature extraction from raw iEEG data, and power envelopes from several frequency bands as additional input to the CNN. In order to prevent mutual misclassification of artifacts and pathological activity (typically interictal epileptiform spikes and high-frequency oscillations), we also trained the CNN for classification of segments that were manually classified to gold standard group as pathological local field potentials without specifying any pathology type. However, classification of specific pathological segments is possible and will be the subject of further research.

The results from Tables 2 and 3 describe the system behaviour on completely unseen data from a different institution. Those results represent worst case scenario when patients’ data are obtained from different acquisition systems. Moreover, the model was tested on patients likely with different behavioural states (awake vs sleep), which also have different characteristics. Results show low mutual misclassification between noise and pathological iEEG, this successfully fulfils the requirement for further HFO analysis. On the other hand, mutual misclassification between physiological iEEG and pathological iEEG is higher, but in expected range (not purpose of proposed detector). This observation is reasonable regarding a fact that boundaries between physiological and pathological iEEG cannot be clearly defined in all the patients and there is also high inter-rater variability for these two classes in ranking real data.

We suggest that retraining of a generalized CNN model to create a data specialized model is needed when a significant change in signal properties is introduced, for example by using different electrode types, acquisition system, or power line frequency. This yield in significantly better performance (F-score 0.96) using only minimal data for re-training.

The generalized CNN may be used to preliminarily score the new data, double checked by a visual inspection and manual corrections for new training samples. A drawback of this method is the dependence on sampling frequency due to direct feature extraction by convolution filter layers from raw data, but this could be solved by data resampling.

In conclusion, this paper introduces an iEEG noise detection technique based on CNN and a graphical display to interpret and analyze results using Artifacts Probability Matrix and Pathology Probability Matrix. The results demonstrate that CNN can provide automated artifact identification in iEEG. We have shown that the generalized CNN model can be used with promising results to classify iEEG data among multiple research institutions. The numerical results allow for subsequent automatic processing and evaluation of the actual biological and pathological deep brain activity.

## Information Sharing Statement

In order to promote the reproducibility, replicability, generalizability and reusability of our research we published the codes for training of neural networks at: https://github.com/xnejed07/NoiseDetectionCNN. Overall size of the used data exceed several hundred of gigabytes, can not be publicly shared and requires special functions for decompression and reading (multiscale electrophysiology format (.mef) and d-file(.d)). However, data might be obtained upon request by contacting principal investigator of the projects. Mayo clinic data may be obtained through the web site of professor Gregory Worrell, M.D., Ph.D (http://msel.mayo.edu) and St. Anne’s data might be obtained by contacting professor Milan Brazdil, M.D., Ph.D.
